# Updated Canine Leishmaniosis Working Group recommendations for leishmaniosis in dogs: Q&A on clinical management

**DOI:** 10.1186/s13071-026-07446-6

**Published:** 2026-05-29

**Authors:** Xavier Roura, Saverio Paltrinieri, Oscar Cortadellas, Alessandra Fondati, George Lubas, Eric Zini, Domenico Otranto, Silvia Lucia Benali, Andrea Zatelli

**Affiliations:** 1 Hospital Clínic Veterinari, Universitat Autònoma de Barcelona, Bellaterra, Spain; 2 Department of Veterinary Medicine and Animal Sciences, University of Milan, Lodi, Italy; 3 Hospital Clínico Veterinario, Universidad CEU Cardenal Herrera, Valencia, Spain; 4 Veterinaria Cetego, Rome, Italy; 5 Clinica Veterinaria Colombo, VetPartners Italia, Lucca, Italy; 6 Department of Animal Medicine, Production and Health, University of Padova, Legnaro, Italy; 7 Clinic for Small Animal Internal Medicine, Vetsuisse Faculty, University of Zurich, Zurich, Switzerland; 8 Department of Veterinary Medicine, University of Bari, Valenzano, Italy; 9 Department of Veterinary Clinical Sciences, City University of Hong Kong, Hong Kong, China; 10 MYLAV Veterinary Laboratory, Milan, Italy

**Keywords:** *Leishmania infantum*, Diagnosis, Treatment, Prevention

## Abstract

Canine leishmaniosis is an important vector-borne disease and a threat to dogs worldwide. Disease data are continuously being collected, with a consequent increase in the number of publications. This article provides practical guidelines for veterinary practitioners on the diagnosis, treatment, and prevention of leishmaniosis in dogs. These Canine Leishmaniosis Working Group (CLWG) recommendations aim to provide practical answers to the most common questions on the clinical management of this disease.

**Graphical Abstract:**

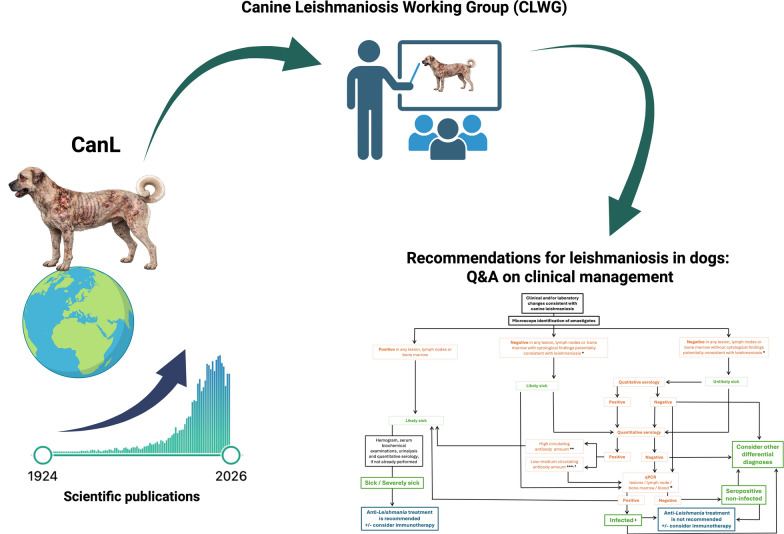

## Background

The Canine Leishmaniosis Working Group (CLWG) was established in November 2005 to develop a science and evidence-based consensus for the diagnosis and management of leishmaniosis in dogs. The CLWG has since published several papers on the diagnosis, clinical classification, treatment, prognosis, and prevention of this disease [1–6].

The primary goal of the CLWG recommendations is to support veterinary practitioners in diagnosing and managing canine leishmaniosis (CanL) and to bring together and standardize diagnostic, therapeutic, and preventive approaches to this zoonotic disease caused by the protozoan *Leishmania infantum* [3, 7, 8]. These updated CLWG recommendations include dogs in all disease stages, namely, seropositive non-infected, infected, sick, and severely sick (Table 1). They are based on published evidence and/or the clinical experience of participating members in the last 15 years since the CLWG’s first publications on clinical staging [2, 3].

**Table 1:** Updated canine leishmaniosis clinical staging system of CLWG

Stage	Definition	Description
A	Seropositive non-infected	Dogs that are either clinically normal or with clinical and/or clinicopathological abnormalities not associated with leishmaniosis, who live or have lived during at least one transmission season in a geographical area where sand flies are endemic. *Leishmania* parasites cannot be detected by qPCR. Typically, a low quantity of circulating *Leishmania*-specific antibodies, but rarely, also medium or high
B	Infected	Dogs that are either clinically normal or with clinical and/or clinicopathological abnormalities not associated with leishmaniosis or active infection. * Leishmania* parasites can be detected by qPCR on bone marrow, lymph node, spleen, skin, or conjunctive samples. Typically, negative or low, but rarely, also medium or high quantity of circulating *Leishmania*-specific antibodies
C	Sick	Dogs with clinical signs and/or clinicopathological abnormalities associated with leishmaniosis or with active infection. *Leishmania* parasites can be demonstrated, by microscopy or qPCR. Typically, high or medium, but rarely, also negative or low quantity of circulating *Leishmania*-specific antibodies. Although this is not a clinical option recommended by the CLWG, very exceptionally, dogs presenting for the first time with clinical signs and/or clinicopathological abnormalities compatible with leishmaniosis, and with a high quantity of circulating *Leishmania*-specific antibodies of ≥ 3 dilutions (IFAT) or ≥ threefold (ELISA) the cutoff value used by the laboratory to define a sample as positive, could be treated using anti-*Leishmania* drugs even if the presence of the parasite could not be demonstrated using direct diagnostic techniques
D	Severely sick	Sick dogs (stage C) with: (1) severe proteinuria (UPC > 3), (2) severe kidney disease (IRIS stage 3–4*) or (3) severe skin, eye, joint or other disease, which can lead to a serious alteration of its function and/or require immunosuppressive therapy

^*^IRIS staging of CKD in dogs (www.iris-kidney.com)

*qPCR* quantitative polymerase chain reaction, *UPC* urine protein-to-creatinine ratio, *IRIS* International Renal Interest Society, *CKD* chronic kidney disease

The CLWG clinical staging system [3] differentiates between infected and clinically sick dogs, whereas the LeishVet staging system [7] only classifies clinically sick dogs [9]. However, these stages are not permanent and can vary throughout a dog’s life, depending on multiple factors, such as aging, whether it lives in an endemic area, lifestyle, the presence of comorbidities, or treatments used [3, 4]. The CLWG recommendations thus aid clinicians in understanding whether a dog is truly sick (suffering from leishmaniosis) and therefore requires anti-*Leishmania* treatment. This approach helps mitigate overtreatment, thereby reducing the potential for the emergence of drug resistance [9]. Additional positive implications include lower costs for owners and the reduced risks of adverse side effects in the treatment. Note that clinicians are encouraged to critically assess the potential applicability of the clinical staging system and the CLWG’s recommendations when managing dogs with leishmaniosis. The specific epidemiological and clinical context should always be considered, as well as the legal regulations of the country in which veterinarians operate in terms of therapy and notification requirements.

These CLWG recommendations have been unanimously agreed upon and approved by all members of the group. The aim is to summarize the diagnosis, treatment, and prevention of CanL to help veterinary practitioners in managing this disease. Although these updated CLWG recommendations are focused on Old World CanL, many of the topics discussed can also help veterinary practitioners working with New World CanL.

### Classification of dogs as seropositive non-infected, infected, sick, and severely sick

#### 1. When should a dog be considered as seropositive non-infected?

The definition of this clinical stage, which was originally outlined as exposed in the previous CLWG clinical staging [2, 3], has now been modified and referred to as the seropositive non-infected stage.

Seropositive non-infected dogs (stage A; Table 1) reside or have lived for at least one transmission season in a geographical area where sand fly vectors and protozoa are present, and are identified by:

- i. Typically, a low quantity of anti-*Leishmania* antibodies, but rarely, also medium or high. The numbers are obtained using quantitative tests, immunofluorescence antibody test (IFAT) and enzyme-linked immunosorbent assay (ELISA), which are preferred over qualitative tests given the low sensitivity of the latter when the number of circulating antibodies is low [2, 3, 10, 11] (see also questions 11–13).
- ii. Negative polymerase chain reaction (PCR), from now quantitative PCR (qPCR), is essential [12]. This is performed on samples that are typically characterized by hosting a high number of parasites such as bone marrow, lymph node, spleen, skin, or conjunctiva [13, 14]. Negative microscopic examination of these specimens does not rule out the presence of *Leishmania* amastigotes, given the low sensitivity of this technique with low parasite numbers (see also question 10).
- iii. No clinical signs on physical examination and no clinicopathological alterations on laboratory tests, including complete blood cell count (CBC), serum biochemical profile, serum protein electrophoresis (SPE), and urinalysis, attributable to *Leishmania*. However, seropositive non-infected dogs can have clinical and/or clinicopathological signs associated with other diseases, possibly mimicking leishmaniosis. The exclusion of infection is therefore crucial when diseases need to be treated long term with immunomodulatory–immunosuppressive drugs (see also questions 7 and 8 and Table 2).

**Table 2:** General and specific clinical findings in dogs suffering from leishmaniosis

Location	Clinical findings
General	Poor nutrition state up to cachexia Mild-to-severe muscular atrophy Lethargy Pale mucous membranes Mild-to-moderate enlargement of palpable lymph nodes Epistaxis Hepatosplenomegaly Lameness and joint swellings Fever
Cutaneous and mucocutaneous	Exfoliative dermatitis (localized/generalized) Ulcerative dermatitis with varying appearance and distribution Mucocutaneous junctions Skin covering the extremities Traumatized sites Papular dermatitis Nodular dermatitis Lupus/pemphigus-like nasal lesions Onychopathy (with onychogryphosis as more typical nail lesion) Nasodigital hyperkeratosis Pustular dermatitis
Ocular	Palpebral lesions: see cutaneous and mucocutaneous findings Diffuse and/or nodular conjunctival lesions Corneal lesions, mainly associated with the conjunctiva (keratoconjunctivitis) Nodular keratitis and keratoconjunctivitis sicca Scleral lesions: diffuse and/or nodular scleritis and episcleritis Diffuse and/or granulomatous lesions of anterior uvea and lesions of posterior uvea (chorioretinitis, hemorrhages, and retinal detachments) Possible complications of uveal diseases are glaucoma and panophthalmitis Granulomatous orbital lesions, myositis of extrinsic muscles
Others	Gastrointestinal, neurological, or bone involvement

Seropositive non-infected dogs do not need to be treated with anti-*Leishmania* drugs. However, there are currently no prospective longitudinal studies specifically focused on these dogs. This heterogeneous stage may include infected dogs that remain unidentified owing to the low parasite burden (undetectable with the available techniques), possibly localized in untested substrates [15]. Seropositive non-infected dogs should therefore be monitored at least once a year. If the number of circulating antibodies increases and/or clinicopathological alterations suggestive of active infection (see also question 4) are noted over time [16], direct diagnostic tests should be repeated to rule out *L. infantum* infection, even when there are no clinical signs.

Finally, seropositive non-infected dogs can also include dogs with anti-*Leishmania* circulating antibodies due to nonspecific reactions, for example, cross-reaction with other *Leishmania* species such as *Leishmania tarentolae* [17, 18] or *Leishmania tropica* [18, 19], or dogs with spontaneous transient infection [15, 20–22]. Depending on the underlying cause of seropositivity, the quantity of circulating anti-*Leishmania* antibodies may remain low chronically, fluctuate, or spontaneously turn negative. For example, nearly a quarter of clinically healthy seropositive dogs living in an endemic area become seronegative by the end of the next nontransmission season [23, 24].

#### 2. When should a dog be considered as infected?

Infected dogs (stage B; Table 1) typically exhibit:

i. Serology typically negative or low, but rarely also medium or high quantity of circulating anti-*Leishmania* antibodies [2, 3, 10, 11] (see also questions 11–13).
ii. Positive qPCR [12] performed on samples typically harboring high parasite loads such as bone marrow, lymph node, spleen, skin, or conjunctiva [11, 13, 14] (see also question 10).
iii. Good health or clinical and/or clinicopathological alterations on physical examination and laboratory tests (including CBC, serum biochemical profile, SPE, and urinalysis) not associated with leishmaniosis or with active infection (see also questions 4, 7, and 8 and Table 2).

However, the interpretation of the number of circulating anti-*L. infantum* antibodies and detection of *Leishmania* DNA by qPCR require caution, particularly in relation to the vector/transmission periods. In endemic areas, the number of antibodies can increase [23, 24], and qPCR assay can be positive in skin or conjunctival samples during the transmission season [13]. This means that it can be challenging in dogs from an endemic area, with compatible clinical signs and/or clinicopathological changes compatible with leishmaniosis and positive serology, to confirm whether they are sick or are simply infected but suffering from another disease that mimics leishmaniosis.

For example, to differentiate infection from disease in dogs with dermatological alterations, the presence of parasites in lesional sites needs to be verified [25, 26]. In these dogs, a positive qPCR in the bone marrow or lymph node would not differentiate infection from disease, as it could be an infected dog with a concurrent skin disease that mimics leishmaniosis. In cutaneous samples, direct techniques should be used in ascending order of diagnostic sensitivity, from cytology–histopathology to immunohistochemistry and to qPCR [26].

Importantly, the infected stage also includes dogs that transitioned from the sick to the infected stage after anti-*Leishmania* treatment. In this case, mild-to-moderate clinicopathological changes are usually detected because a state of immune response and an inflammatory process persist due to stimulation by a latent low *Leishmania* load [23, 24]. Positive serological and molecular diagnostic tests should therefore be evaluated carefully. Each dog’s clinical situation needs to be considered, because mild-to-moderate clinicopathological changes in an infected dog do not necessarily indicate a relapse (see also question 4).

From a clinical point of view, infected dogs do not need to be treated with anti-*Leishmania* drugs, even with a stable high quantity of circulating anti-*Leishmania* antibodies or if the number of antibodies increases within a few weeks after the first serological diagnosis (see also questions 7 and 8). These cases should be serologically monitored at varying intervals, depending on the quantity of circulating antibodies present at the previous check-up. This means that with a positive serology result and a high number of antibodies, a subsequent check-up is recommended after 2–4 months. However, with a positive serology result but with few antibodies, the next check-up is recommended in 6–12 months [2, 4] (see also question 28).

#### 3. When should a dog be considered sick?

Sick (stage C) and severely sick (stage D) dogs (Table 1) suffer from leishmaniosis and are characterized by:

i. Clinical signs on physical examination and/or clinicopathological abnormalities on laboratory tests, including CBC, serum biochemical profile, SPE, and urinalysis, attributable to leishmaniosis (see also questions 2, 4, 7 and 8 and Table 2).
ii. *Leishmania infantum* infection should be demonstrated by direct diagnostic tests, microscopy, or qPCR. Since the quantity of parasites present in dogs affected by leishmaniosis is usually high, it could be possible to demonstrate their presence with microscopic techniques. However, sometimes qPCR could be necessary to demonstrate the presence of *Leishmania* in these dogs. Culture is also a direct diagnostic technique, but it is not useful in clinical practice [11] (see also questions 9 and 10).
iii. Frequently positive with medium or high value of circulating *Leishmania*-specific antibodies in quantitative tests, IFAT and ELISA, but occasionally also low amount or negative [2, 3, 10, 11] (see also questions 1, 2, and 11–13).

From a clinical point of view, sick-staged dogs need to be treated with anti-*Leishmania* drugs, and the prognosis is favorable to guarded depending on the severity of clinical signs and clinicopathological changes, particularly those related to the kidney [6]. Although this is not a clinical option recommended by the CLWG, very exceptionally, dogs presenting for the first time with clinical signs and/or clinicopathological abnormalities compatible with leishmaniosis, and with a high quantity of circulating *Leishmania*-specific antibodies, could be treated using anti-*Leishmania* drugs even if the presence of the parasite could not be demonstrated using direct diagnostic techniques [2, 3, 11]. For the vast majority of laboratories or diagnostic kits, a high quantity of *Leishmania*-specific antibodies is defined as a high quantity of *Leishmania*-specific antibodies, which is defined as ≥ 3 dilutions (IFAT) or ≥ threefold (ELISA) the cutoff values used by the laboratory to define a sample as serologically positive. Therefore, this means a result of ≥ 1/320 dilution if the cutoff value is 1/40 or a result of ≥ 1.65-fold if the cutoff value is 0.55, when using IFAT or ELISA, respectively (see also question 11).

Different stages of sickness (i.e., sick and severely sick) depend on the severity of clinical and clinicopathological changes due to immune complex deposition in the kidney and in extrarenal sites, including the skin, eyes, and joints, and the respective therapeutic challenge associated with the use of immunosuppressants along with anti-*Leishmania* treatment [2, 7]. In these dogs, the prognosis is reported as guarded to poor.

#### 4. When does a dog have an active infection?

The term “active infection” refers to the progression from the infected to the sick stage. Dogs with an active infection are thus suffering from leishmaniosis and need anti-*Leishmania* treatment. Leishmaniosis in dogs is considered a latent infection with a low parasitic load; however, a certain percentage of dogs in the infected stage will progress to sick stages, months to years after contracting the infection. This can happen, for example, if the dog develops another comorbidity or receives therapies that can disrupt its immune response, or in the case of relapse after anti-*Leishmania* treatment (Table 1) [4]. It is very important to differentiate between the two stages, infected and sick, because in the infected stage, the dog does not need anti-*Leishmania* treatment, while in the sick stage, the dog needs treatment [2].

As previously mentioned, it is relatively easy for veterinary clinicians to differentiate between infected and sick stages at the first diagnosis. However, diagnosis becomes more complicated when a dog has concomitant diseases that may cause clinical or clinicopathologic changes that overlap with those of leishmaniosis (e.g., certain chronic inflammatory skin diseases or concurrent vector-borne diseases).

It is even more difficult to differentiate between infected and sick stages in the case of a relapse after anti-*Leishmania* treatment. This is because latently infected dogs, after treatment, commonly maintain different degrees of clinicopathological alterations associated with the dog’s inflammatory–immune response to *Leishmania*. These alterations, therefore, do not necessarily indicate a relapse. Moreover, there are no perfect diagnostic tests that differentiate between infected and sick dogs [16, 23, 24].

The initial transition from infected to sick stage (active infection) is invariably characterized by an increased local or systemic parasitic load, which makes it easier to detect *Leishmania* in dogs with sick stage compared with infected ones. From a clinical point of view, the demonstration of *Leishmania* by microscopy in specific lesions, bone marrow, and/or lymph node, or by qPCR in specific lesions and/or whole blood [14], is a practical way to differentiate infection from active-infection sickness. However, both in the case of infection and in active-infection sickness, ruling in or ruling out the presence of *Leishmania* by direct diagnostic techniques before making decisions on anti-*Leishmania* treatment is still strongly recommended. This is because:

i. Leishmaniosis in dogs is characterized by clinical and histopathological pleomorphism, a lack of clinicopathological specificity and variable seropositivity [3, 11].
ii. The prevalence of infection in endemic areas is higher than both the seroprevalence and prevalence of disease [27].
iii. Seropositivity might be due to cross-reactions with other *Leishmania* species, including nonpathogenic ones, such as *L. tarentolae* [28].

### Diagnosis

#### 5. How can we make a reliable diagnosis of CanL?

**Fig. 1:**
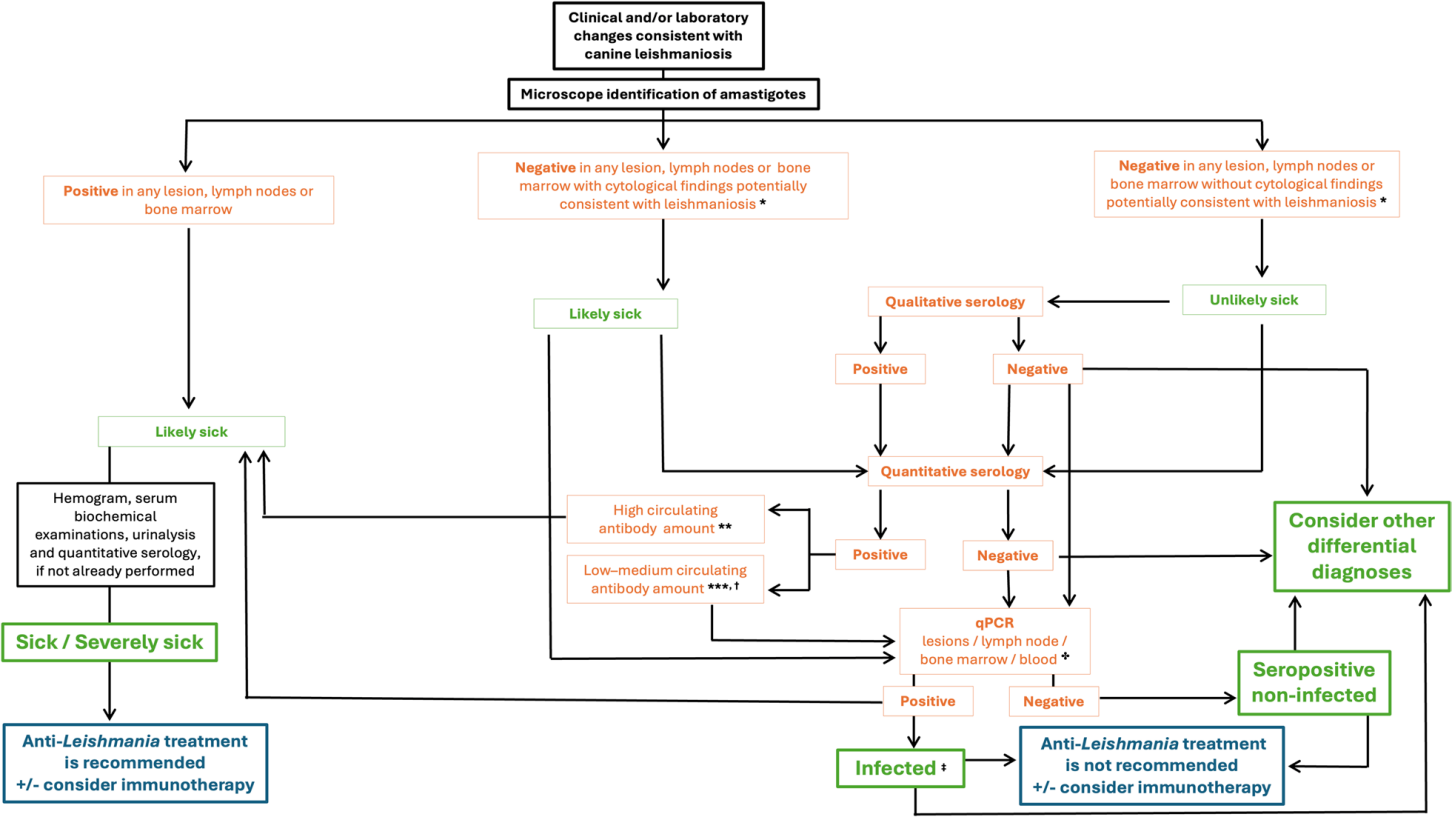
Flow chart of diagnosis, clinical staging and treatment recommendations for dogs suspected of suffering leishmaniosis. * Macrophages and neutrophils in lesions; reactive/hyperplastic lymph nodes; bone marrow with myeloid hyperplasia and/or erythroid hypoplasia, possibly associated with plasmocytosis. ** Both IFAT ≥ 3 dilutions or ELISA ≥ threefold the cut-off value used by the laboratory to define a sample as positive. Consider possible cross-reactivity with other species where are present, e.g., *L. tarentolae*. *** Both IFAT 3 dilutions or ELISA threefold the cut-off value used by the laboratory to define a sample as positive. Consider possible cross-reactivity with other species where are present, e.g., *L. tarentolae*. † Low-medium antibody amount in the transmission season might depend on seroconversion after sandfly bites. Carefully interpret these results and/or repeat testing outside the transmission season. ^†^ Blood is not an adequate sample for PCR for first diagnosis due to low sensitivity; but it could be an adequate sample for PCR to diagnose a potential relapse. ‡ The PCR positivity in dogs with low antibody amount indicates infection, but the likelihood of active infection-sickness increases if cytology is consistent with leishmaniosis despite the lack of visible amastigotes

Although a flowchart (Fig. 1) cannot include all the different aspects of a disease as complex as CanL, it provides a practical summary of the diagnostic protocol. The diagnosis that a dog is suffering from leishmaniosis must be based on the combination of information on the exposure to the parasite, the detection of suggestive clinical signs and/or clinicopathological alterations (Tables 2 and 3), confirmation of the infection, and a serological assessment of the immune response [11, 29]. Thus, to detect alterations indicative of CanL, in addition to a detailed clinical history, anamnesis, and physical examination, a diagnostic workup that includes routine laboratory examinations, such as CBC, serum biochemical profile, and SPE; urinalysis with urine protein-to-creatinine ratio (UPC); and any other ancillary tests is needed [11, 29]. The diagnostic workup should be performed even in dogs without clear clinical signs of leishmaniosis. This is because some alterations (e.g., proteinuria or gammopathy) may occur earlier than clinical signs [30, 31]. However, most of the clinical and laboratory findings that characterize leishmaniosis are nonspecific and are also found in other diseases. Therefore, if leishmaniosis is suspected, infection should be confirmed by directly detecting the parasite (e.g., by cytology or histology) or its DNA (e.g., by qPCR) and by using serology to assess the dog’s immune response against the parasite [3].

**Table 3:** Results in basic and advanced laboratory tests consistent with dogs suffering from leishmaniosis

Tests	Findings	Further tests
CBC	Poorly regenerative or nonregenerative anemia (generally mild to moderate, normocytic–normochromic)	
	Occasionally regenerative anemia (due to immune-mediated processes)	Flow cytometry to detect anti-erythrocyte antibodies
	Occasionally neutrophilic and monocytic leukocytosis with lymphopenia and eosinopenia (stress leukogram/inflammation)	
	Reactive lymphocytes (often) Leukopenia	Bone marrow cytology
	Possible mild-to-moderate thrombocytopenia	Extended coagulation profile (e.g., fibrinogen, FDP, AT, and possibly D-dimer)
		Search for coinfection (e.g., with *Ehrlichia* or *Anaplasma*)
	Mild-to-severe alteration of erythrocyte sedimentation rate	Flow cytometry to detect antiplatelet antibodies
Basic coagulation profile	Hyperfibrinogenemia (mild to moderate), possible (mild) increase of PT and aPTT	Extended coagulation profile (e.g., fibrinogen, FDP, AT, and possibly D-dimer)
Serum biochemical panel	Hyperproteinemia, hypoalbuminemia, hyperglobulinemia, albumin/globulin ratio (significantly altered)	APP (CRP, Hp, SAA, ferritin) and iron (significantly altered)
	Azotemia (mild-to-high serum values of urea [BUN] and creatinine)	Lipid parameters: mild-to-moderate hypercholesterolemia
		Electrolytes: mild hypokalemia
		Mild-to-moderate hyperphosphatemia and hypermagnesemia
		Blood gas analysis (mild-to-moderate metabolic acidosis)
	Increased (mild to moderate) hepatic enzymes concentrations	Liver function tests (e.g., bile acids)
SPE	Hypoalbuminemia, increased α_2_-globulins and poly/oligoclonal gammopathy (severely altered)	
Urinalysis	Isosthenuric urine (SG: 1008–1015) or poorly concentrated urine (< 1030)	
	Mild-to-severe proteinuria (determined by dipstick and UPC ratio)	SDS–AGE urine (mild to markedly altered)

*CBC* complete blood count, *FDP* fibrin/fibrinogen degradation products, *AT* antithrombin III, *PT* prothrombin time; *aPTT* activated partial thromboplastin time, *APP* acute-phase proteins, *CRP* C-reactive protein, *Hp* haptoglobin, *SAA* serum amyloid A, *SG* specific gravity, *UPC* urinary protein-to-creatinine ratio, *SDS–AGE* sodium dodecyl sulfate–agarose gel electrophoresis; *SPE* serum protein electrophoresis

To summarize (Fig. 1), the diagnosis of a sick dog must be based on the detection of *Leishmania* amastigotes using direct techniques such as microscopic examination of cytological or histopathological specimens. Otherwise *Leishmania* DNA should be assessed using qPCR, which should be performed when microscopic examination is consistent with leishmaniosis, but no amastigotes are microscopically observed. Finally, very exceptionally, because this is not a clinical option recommended by the CLWG, when the dog with clinical signs and/or clinicopathological abnormalities compatible with leishmaniosis is being diagnosed for the first time, a high quantity of circulating *Leishmania*-specific antibodies may suggest that it is suffering from leishmaniosis even though *Leishmania* parasite cannot be identified by direct diagnostic techniques [2, 3, 11] (see also question 3 and Fig. 1).

#### 6. Is there a genetic innate resistance to *Leishmania* infection?

The evolution of *Leishmania* infection and its clinical manifestations are the result of the complex interactions between the dog’s immune system and the parasite. The current disease model for leishmaniosis suggests that infected dogs can live without progression to clinical disease manifestation, probably due to the immune control of the infection [32]. The factors that determine whether individuals progress to clinical disease following *Leishmania* infection are unclear. However, previous studies have suggested that the host genetic background plays a major role [33, 34].

In dogs, genetic susceptibility to progression of the disease from *Leishmania* infection is supported by the fact that the percentage of infected dogs in endemic areas is as high as 60% [35], whereas in the same areas, rates of clinical leishmaniosis are much lower [36]. Similar to familial aggregation and ethnic differences in clinical leishmaniosis prevalence seen in humans and mice, different dog breeds may be susceptible to leishmaniosis in different ways. Some breeds, such as the boxer, German shepherd, and rottweiler, appear more predisposed to overt leishmaniosis [37–39]. In contrast, the Ibizan hound breed has been reported to be resistant to the development of leishmaniosis [40, 41].

The resistance depends on the immune response, whether innate or acquired (humoral and cellular), that the dog elicits soon after the sand fly bite and the inoculation of *Leishmania* promastigotes [33, 34]. Similarly, the type of immune response seems to be almost 60% determined by the dog’s genetics and about 40% by other factors such as diet, concurrent diseases, coinfections, the drugs used, frequency of exposure to sand flies, etc. [39, 42, 43]. These findings present new approaches for controlling CanL such as gene therapy or the use of immunotherapy [15, 43–46].

#### 7. What are the most reported and/or new clinical signs?

CanL presents a wide variety of clinical signs, not only due to the infection itself but also to how a dog’s immune system responds. In dogs with leishmaniosis, dermatological signs and lymphadenomegaly are the most frequently described, although ocular or renal clinical manifestations may also occur (Table 2) [3, 7–9, 11, 31, 47, 48]. In addition, although many skin, oral, or ocular clinical manifestations in dogs are considered atypical or rare, such as mucocutaneous nodular dermatitis, papular glossitis, or corneal nodules, veterinarians working in endemic areas commonly include leishmaniosis in their differential diagnosis even when unusual findings have been observed [49–56].

In the last decade, additional clinical changes have been reported in dogs. The four most important are chronic enteropathy [57–59], myocarditis [60–62], osteoperiostitis [63–65], and central and peripheral nervous system alterations [66–69]. All these four clinical pictures are characterized by being difficult to diagnose, since around 50% of dogs do not show clinicopathological alterations typical of leishmaniosis. A final confirmation can only be achieved by demonstrating the inflammation associated with *Leishmania* parasites, the latter through conventional histopathology, immunohistochemistry, or qPCR in biopsies of the affected tissue [70–72].

#### 8. What are the most reported and latest laboratory abnormalities?

Frequent laboratory abnormalities include mild-to-moderate normocytic normochromic nonregenerative anemia, thrombocytopenia, hyperproteinemia, hyperglobulinemia, hypoalbuminemia, low albumin-to-globulin ratio, increased creatinine and/or urea, and proteinuria (Table 3) [11, 29]. Detection of increased numbers of α_2_-globulins and a polyclonal gammopathy in SPE are the most frequently reported findings in CanL. However, other chronic inflammatory or vector-borne diseases induce similar profiles (e.g., atopy or ehrlichiosis) and need to be ruled out [73–75]. SPE may help to monitor the treatment success (α_2_-globulins are expected to decrease soon after treatment, gamma-globulins tend to decrease after 1–2 weeks and frequently become normal in 4–6 weeks) or to suggest early identification of potential relapses in treated dogs [4, 76–78].

Another common laboratory finding in dogs with leishmaniosis is an increase in positive acute-phase proteins (APP) such as C-reactive protein (CRP), ferritin, haptoglobin (Hp), or serum amyloid A (SAA), or a decrease in negative APP, such as paraoxonase (PON-1) [16, 79–88]. The erythrocyte sedimentation rate was recently used as an in-clinic assay in CanL. It was markedly affected by the severity of CanL and could be used to monitor treatment efficacy [89–91]. The overall changes presented above cannot be used alone to diagnose CanL, as they are not specific, since they may also be found in any inflammatory condition [16]. However, in dogs with confirmed infection, changes in APP may support the hypothesis that they are suffering from leishmaniosis, although the severity of their increase does not seem to be associated with the severity of the clinical condition, except for PON-1, which decreases only in severe clinical forms [92]. Furthermore, as with SPE, APP may be a useful tool to monitor the course of treatment, since they normalize earlier than SPE (3–20 days, depending on the APP) [80, 84].

Other old and new markers have been proposed to differentiate sick from infected dogs, to stage the severity of the disease, or to provide prognostic information, either based on their concentration at admission or on their normalization during the follow-up. These include: (1) markers of cell-mediated (Th1) versus humoral (Th2) immunity (CD4:CD8 ratio), which, when decreased, may reveal a shift from Th1 (protective) to a Th2 (nonprotective) immunity [93, 94]; (2) markers of tubular damage, which increase when the renal disease transitions from early glomerular damage to a tubule-interstitial nephritis. These include the identification of tubular proteins through sodium dodecyl sulfate electrophoresis (SDS–AGE) and the measurement of gamma-glutamyl transferase (GGT), *N*-acetyl-beta-d-glucosaminidase (NAG), neutrophil gelatinase-associated lipocalin (NGAL), urinary gamma-glutamil transferase (uGGT), clusterin, ferritin, and amylase [95–106]; and (3) molecules involved in the generation of tissue lesions such as circulating immune complexes (CIC), which may differentiate seropositive non-infected or infected dogs from those that are sick [107–111].

#### 9. What does a positive cytology for *Leishmania* mean?

Cytology enables the direct visualization of *Leishmania* within tissues. If the parasitic load is high, amastigotes can generally be identified within macrophages or extracellularly [3, 11].

Cytology is highly specific, so the presence of amastigotes on cytology is crucial for the clinical classification of CanL and for its appropriate treatment. A positive cytology thus confirms the dog’s status not only as infected but also the status of active infection or sickness. A dog is sick (stage C or D) if the parasite is identified in bone marrow or in samples with compatible lesions, such as from skin, enlarged lymph nodes, or biological fluids from affected sites (e.g., joints or eyes) [3, 11].

In contrast, the sensitivity of cytology is low because it relies on the number of amastigotes in the lesions and is dependent on the skills of the pathologist. In some organs (e.g., joint or kidney), tissue lesions are mostly secondary to immune complex deposition and not to the direct presence of the parasites, thus sampling of these tissues rarely detects amastigotes [6, 72, 112].

When amastigotes of *Leishmania* are not observed on cytology, if other tests or clinical signs are still consistent with leishmaniosis histopathology, immunohistochemistry or qPCR can also be used to detect the parasite [3].

#### 10. What does a positive qPCR for *Leishmania* mean?

Although clinical, serological, and clinicopathological findings may be highly consistent with CanL, confirmation of *Leishmania* is recommended before any treatment whenever possible (see also question 3). This can be performed through direct microscopic techniques; however, they are insufficiently sensitive to low parasitic loads (see also question 9). Therefore, qPCR may be mandatory when microscopic techniques are negative for the parasite or when no macroscopic lesions have been identified [3, 14, 29]. This could be particularly interesting for any type of skin lesion where the use of qPCR, in multiple clinical samples such as needle aspirates, swabs, or filter paper impressions, has proven to be very useful in correlating lesions with the presence of *Leishmania* [113, 114], especially in the presence of typical skin lesions, for example, papules, or in cases of cyto-histopathological findings consistent with or suggestive of CanL.

Given that qPCR is widely used, data on the presence/absence of the parasite or on the parasite burden are thus available [12, 14]. However, methods to quantify the results have not been standardized, and there is little information on the variability between different samples on the same dogs or between different aliquots of the same specimen, such as two “drops” of the same bone marrow [115, 116]. Together with the interlaboratory variability that characterizes any test, this hampers a uniform interpretation of the qPCR results or the use of this test in monitoring the parasitological clearance in treated dogs [12, 14].

In addition, qPCR results are affected by the tissue itself. A positive result is highly specific for the detection of infection regardless of the specimen, except for skin lesions where a positive qPCR may depend on the presence of parasites recently inoculated by the sand fly, rather than on a true infection. Conversely, the probability of positive results in infected dogs is relatively high in bone marrow and/or in lymph nodes owing to the high sensitivity of qPCR. If skin, eye, or joint lesions are not present [12, 14], to confirm infection, bone marrow and/or lymph nodes should thus be used as samples. However, sensitivity of the molecular tests may be low in other tissues, including blood, which is frequently negative in infected dogs, or ulcerated skin, on which the inflammation response may have a deleterious effect on the parasite’s DNA [11, 29, 115].

#### 11. What are the main differences between the serological techniques available? Which one is best and why?

Anti-*Leishmania* antibodies are detected using IFAT, ELISA, or rapid immunochromatographic tests (ICT).

The IFAT has long been considered the cornerstone for the serological diagnosis of CanL and serves as the gold standard for detecting anti-*Leishmania* antibodies, as recommended by the World Health Organization and World Organization for Animal Health [117]. However, in both clinical practice and research settings, ELISA and ICT are also widely employed.

Owing to its low sensitivity, the ICT can be used when dogs present clinical signs but should not be used for screening clinically healthy dogs [3, 29, 115, 116]. Moreover, ICT does not provide antibody quantification, which is important information to support clinical diagnosis and monitor the antibody response during follow-up. A decrease in the quantity of circulating antibodies is in fact expected between 3 and 6 months after the start of treatment [76, 118].

ELISA or IFAT are thus preferred owing to their higher sensitivity and because they quantify the antibody response. IFAT used to be considered the gold standard test since it is more sensitive than ELISA. However, the sensitivity of modern ELISA kits is comparable to that of IFAT [119], and ELISA is therefore preferred in practice, as it is cheaper than IFAT, less time-consuming, and less operator-subjective in the interpretation of the results. If ELISA results are negative in dogs with a high likelihood of infection based on clinical or epidemiological data, it may be advisable to repeat serology using (i) another type of ELISA or (ii) an IFAT method.

Semiquantitative ELISAs also exist. Rather than quantifying the number of antibodies, they compare the intensity of the dog’s positivity with the intensity of a positive control. The results are thus expressed as a number, rather than a real quantity. The stronger the difference between the intensities of the positivity, the higher this number will be, and therefore, the greater the quantity of circulating antibodies [120, 121].

#### 12. How should a positive IFAT and a negative ELISA result be interpreted?

Occasionally, there are discrepancies between IFAT and ELISA, the two most used serological tests, particularly in regions where other trypanosomatids (e.g., *Trypanosoma cruzi*) or *Leishmania* species (e.g., *Leishmania braziliensis*, *Leishmania major*, or *Leishmania tarentolae*) are endemic [17, 120–124]. Although both tests are generally characterized by a high sensitivity (albeit rarely reaching 100%), cross-reactivity with antibodies against other pathogens may compromise their specificity. For example, in a study conducted in southern Italy, a CanL-endemic area where *L. tarentolae* is also found, IFAT revealed the highest proportion of seropositivity (85.6%), probably due to the higher sensitivity and lower specificity of IFAT compared with all the other tests used (two commercial ELISA kits and one in-house ELISA) [17]. Similarly, cross-reactivity of IFAT was reported in dogs in Brazil, where *L. infantum* and *L. braziliensis* infections are endemic [122]. The discriminating factor in accuracy appears to be the type of antigen employed by serological techniques. In fact, different antigen types can be used with ELISA (e.g., whole or soluble extracts of promastigotes, whole or soluble extracts of amastigotes, recombinant proteins, and purified proteins), some of which (e.g., rk39 recombinant protein) may enhance its specificity. Unfortunately, tests that can differentiate between the seropositivity of the different species of *Leishmania* are not commercially available.

An additional issue is that although IFAT may be positive, ELISA may be negative since the ELISA plates may be coated with specific *L. infantum* antigens, while the promastigotes on the IFAT slides also expose antigens that are present in parasites other than *L. infantum*.

Finally, IFAT is an operator-dependent method, so the result may be influenced by the subjective interpretation of the operator, who evaluates the fluorescence of the sample on the basis of their skills and prior experience [125]. In contrast, the results of ELISA plates are assessed by a dedicated spectrophotometer reader, thus reducing the bias due to human interpretation. However, for both serological techniques, technical and laboratory mistakes (analytical errors) should never be excluded.

#### 13. When and how should serology be performed to diagnose CanL?

Quantitative serology should be performed when CanL is suspected on the basis of compatible clinical and/or laboratory data, since usually the higher the antibody response, the greater the likelihood that the dog is sick [3]. This is especially important for the initial diagnosis in endemic areas, where most dogs are seropositive but have a low quantity of circulating antibodies. However, a medium–high amount is more consistent with active infection or with a dog already suffering from leishmaniosis. Consequently, serology must be interpreted on the basis of the region and sampling season.

In endemic regions, during the sand fly transmission season, the number of circulating *Leishmania*-specific antibodies may be high in dogs, probably due to repeated bites from infected sand flies, followed by a decrease to a low number of circulating antibodies or by a negative result, during the nontransmission season [23, 24]. Positive serology should therefore be considered carefully in endemic regions since it may reflect normal seasonal fluctuations rather than an infection or disease status. Conversely, positive serology could be highly suggestive of an active infection or suffering from leishmaniosis in regions with low endemicity or outside the transmission season. Furthermore, in dogs living in or traveling to regions where other trypanosomidae are present (*Trypanosoma cruzi* or *Leishmania tarentolae*), information about these geographic areas should also be considered when interpreting positive results [28, 122] (see also question 12).

Conversely, the current vaccines on the market do not interfere with serology, since the commercially available diagnostic kits do not detect the antibodies induced by the vaccine (see also question 14). Therefore, seroconversion in vaccinated dogs depends on the occurrence of new infections.

Finally, serology may also be performed in clinically healthy dogs potentially exposed to the infection (e.g., after traveling in endemic areas) provided that the test is performed between 2 and 5 months after the exposure (the minimum average time required to seroconvert) [126] and bearing in mind the limitations mentioned above in the interpretation of results (see also questions 11 and 12).

#### 14. Does vaccination interfere with routine serological testing?

Current vaccines for CanL, in addition to inducing an immune response that limits the progression of the infection, should not interfere with the disease diagnosis. This means that antibodies produced in response to the vaccine should not interfere with routinely employed diagnostic serologic tests. Any interference, in addition to being a diagnostic problem, may also have a negative impact on CanL surveillance studies.

CaniLeish^®^ (Virbac, France), the vaccine for CanL used in Europe until 2021 [127], consisted of purified excreted/secreted proteins of *L. infantum* and adjuvant QA-21, a highly purified fraction of saponin (*Quillaja saponaria*). However, CaniLeish^®^-induced antibodies cross-reacted with the serological tests commonly used to diagnose *L. infantum* natural infection and could be detected for up to 1 year after vaccination [128] by quantitative diagnostic tests (IFAT and ELISA) [129–132]. Thus, this made qPCR combined with quantitative serological tests necessary to correctly identify sick dogs [128, 133]. This is no longer a problem because the marketing authorization of CaniLeish^®^ was withdrawn by the European Commission in October 2023 at the manufacturer’s request [127].

With the two vaccines now on the market in Europe, LetiFend^®^ (LETI, Spain) and Neoleish^®^ (CZ Veterinaria, S.A. Spain), no interferences with diagnostic serological tests have been reported to date.

The LetiFend^®^ vaccine has been licensed in Europe since 2016 for the immunization of non-infected dogs. It is a recombinant vaccine composed of a chimeric protein (protein Q) formed by the genetic fusion of four highly antigenic *L. infantum* proteins (histone H2A and three ribosomal proteins LiP2a, LiP2b, and LiP0) with no adjuvants. Vaccination with LetiFend^®^ reduces the quantity of CIC, which is probably related to the mechanism of control of infection in dogs [134] and has not elicited positive results in *L. infantum* serological diagnostic tests (IFAT and soluble *Leishmania* antigen [SLA] ELISA), even in field trials [135].

The Neoleish^®^ vaccine is a nasal spray solution currently (August 2025) marketed only in Spain for the immunization of non-infected dogs. It is a DNA vaccine based on the nonreplicative plasmid vector pPAL coding for the *L. infantum* activated protein kinase C receptor analog (LACK) [136]. Preclinical studies show that Neoleish^®^ seems to elicit a humoral response detected by SLA ELISA characterized by high IgG2 and low IgG1 amounts, which suggests a T cell response skewed toward the Th1 profile [136]. In a report by the Committee for Veterinary Medicinal Products dated 11 November 2022 (EMA/CVMP/858971/2022), Neoleish^®^ was reported not to “interfere with serological diagnostic tools for leishmaniosis, as infected dogs can be distinguished from vaccinated animals and be identified in the population.” No field studies have been published to date.

Based on the above data, if circulating anti-*Leishmania* antibodies are detected in dogs vaccinated with LetiFend^®^ or Neoleish^®^, these should therefore be associated with the parasite rather than the vaccine.

### Treatment

#### 15. When should a dog be treated for leishmaniosis?

In CanL, clinical staging [3, 7] is imperative both at the time of diagnosis and when detecting relapses during the follow-up, since it assists the clinician in deciding whether or not the dog should be treated [2–4, 7, 8] (Fig. 1).

Increasing drug resistance in parasites belonging to the genus *Leishmania* in both human and veterinary medicine is recognized as a major One Health concern [137]. Additionally, several moderate-to-severe side effects have been described in dogs treated for leishmaniosis, and the cost and compliance with anti-*Leishmania* treatment are challenging for owners [6, 138–143]. Misuse of recommended CanL treatment protocols is considered one of the most important factors in the development of adverse effects or resistance [144]. Therefore, it is appropriate to introduce a new concept in the updated CLWG clinical staging, active infection, which defines when the use of CanL treatment is appropriate [3, 4, 6, 16] (see also question 4). Hence, according to this new clinical staging system, practitioners should use anti-*Leishmania* treatment when dogs are in the phase of active infection. Dogs with clinical signs and/or laboratory findings associated with leishmaniosis, categorized as stages C (sick) and D (severely sick), should also be treated. Conversely, anti-*Leishmania* drugs should not be used in dogs in stages A (seropositive non-infected) or B (infected) (Table 1). In general, it is therefore not recommended to use anti-*Leishmania* treatment in a dog solely because the serology test is positive, even if the number of antibodies is very high or increases between two consecutive evaluations (seroconversion).

#### 16. What are the goals of anti-*Leishmania* treatment in sick dogs?

The clinician must choose the best treatment for each individual dog on the basis of clinical presentation, scientific evidence, factors related to the owners and dogs, and each country’s specific legal regulations. However, for sick dogs with leishmaniosis, the most widely recommended and effective treatment protocols are a combination of anti-*Leishmania* drugs, usually 1 month of meglumine antimoniate or miltefosine, together with allopurinol for at least 12 months [2, 6–8, 143, 145].

The current recommended protocols for the treatment of sick dogs reduce the parasite load to meet four key objectives: (1) to decrease or resolve clinical signs, lesions, and laboratory alterations associated with leishmaniosis [2, 7, 8]; (2) to help in restoring an effective immune response aimed at better infection control [146–150]; (3) to prevent relapses by improving the dog’s immune resistance to leishmaniosis, thus keeping the parasite load as low as possible [4, 76, 78, 118]; and (4) to reduce infectivity from sand flies (i.e., the risk of transmitting the infection) among dogs, humans, and other animal species [1, 145, 151, 152].

#### 17. What is the current anti-*Leishmania* treatment protocol recommended for sick dogs?

The most widely used treatment protocol for dogs with active infection or sick is a combination of meglumine antimoniate and allopurinol. Meglumine antimoniate is administered at 100 mg/kg, subcutaneously, once a day for 4 weeks, and allopurinol at 10 mg/kg, orally, every 12 h for 12 months. The dosage of meglumine antimoniate can be divided into two equal doses of 50 mg/kg every 12 h. With this treatment protocol, most dogs achieve stable clinical and laboratory remission for over 1 year, and dogs with a severe form of leishmaniosis have a good chance of clinical improvement [76, 143, 153–158]. If a dog has severe kidney disease prior to the start of treatment, does not respond after treatment with the above protocol, has a relapse (during or after above treatment), develops severe adverse effects (see also question 19), or the owners show poor compliance with drug administration, an alternative protocol could be needed.

An alternative regimen that could lead to fewer adverse effects and improve the owners’ compliance includes miltefosine administered for 28 days (2 mg/kg, orally, once a day) in combination with allopurinol for 12 months (10 mg/kg, orally, every 12 h) [100, 143, 149, 152, 159–164]. However, owing to the risk of earlier relapses or the development of parasite drug resistance, combining miltefosine with allopurinol is considered a second-choice treatment compared with meglumine antimoniate combined with allopurinol [78, 87, 165, 166].

Finally, the old recommendation to treat CanL with allopurinol alone for 12 months (10 mg/kg, orally, every 12 h) is currently generally discouraged because it is much slower to obtain clinical improvement and because it can predispose to the development of resistance to anti-*Leishmania* drugs [144, 150, 158, 165].

Other drugs, such as artesunate, marbofloxacin, intralesional meglumine antimoniate, or sesquiterpene (−)-α-bisabolo, need further research before being recommended as therapeutic options in dogs with leishmaniosis [167–169].

Amphotericin B in lipid emulsion and aminosidine have also been used to treat CanL; however, their use is not recommended as an alternative treatment. This is to prevent the development of resistance in human medicine and toxicity in terms of renal function, respectively [159, 170–172].

#### 18. Are there specific treatment protocols for sick dogs with renal disease?

Managing these dogs requires treating both leishmaniosis and the associated kidney disease. Regarding leishmaniosis, one of the standard anti-*Leishmania* treatment protocols described above is recommended (see also question 17).

The traditional assumption regarding meglumine antimoniate nephrotoxicity has led many clinicians to reject its use or to use it at lower than recommended doses when renal disease is present. However, there is evidence that meglumine antimoniate nephrotoxicity, if it exists, is neither clinically nor clinicopathologically significant [173, 174]. It could be argued that the dose adjustment has been advised for drugs cleared by glomerular filtration, such as meglumine antimoniate and allopurinol, when the glomerular filtration rate is reduced by more than two-thirds [175]. However, the use of these drugs at the standard dose in dogs with leishmaniosis is generally considered safe [6, 160, 176]. Note that using lower than recommended doses could result in reduced treatment efficacy, an increased relapse rate, and treatment resistance [144, 156, 177, 178].

Regarding kidney disease, dogs must be staged, treated, and monitored in accordance with International Renal Interest Society (IRIS) guidelines, bearing in mind that dogs with chronic kidney disease should be staged once the diagnostic markers have been measured at least twice in a normohydrated and stable dog (IRIS guidelines). Although robust evidence is lacking for or against the use of immunosuppressive drugs in dogs with immune complex-mediated glomerulonephritis (the main cause of renal disease in CanL), some experts recommend using glucocorticoids at anti-inflammatory dosages or mycophenolate mofetil to reduce inflammation secondary to deposition of immune complexes [6].

Finally, domperidone or dietary nucleotides with active hexose dietary compound (AHCC) could be used to treat dogs with leishmaniosis and kidney disease. Although the evidence is still sparse, current data indicate that in addition to its immunomodulatory effects in the control of CanL [15, 158], this treatment may also protect the kidney [158, 179, 180].

#### 19. What are the most common side effects of anti-*Leishmania* treatment in dogs?

Although the recommended anti-*Leishmania* treatments can be effective in controlling the disease in dogs (see also question 17), they also have some side effects that can be serious [140, 142, 143, 181].

The most common side effects of meglumine antimoniate include apathy, anorexia, vomiting, diarrhea, pain at the site of injection, and idiosyncratic skin reactions [142]. Approximately half of dogs with side effects require treatment suspension [142, 143]. Increased liver transaminases due to transient hepatotoxicity in the absence of clinical signs have also been described [182], and acute pancreatitis associated with meglumine antimoniate treatment has been reported [182–184], probably due to individual predisposition or unknown concomitant factors [142]. Contrasting concerns regarding meglumine antimoniate on renal function have been raised, with some authors concerned about potential impairment, while others have suggested no renal involvement [6, 142]. Therefore, to minimize some of these adverse effects, it may be advisable to change the skin site with each injection and massage the area carefully and add prednisone at an anti-inflammatory dose (0.7 mg/kg/day) for 5–7 days. However, the frequency and severity of these adverse side effects need further investigation, and it is often difficult to assess whether they are related to the infection itself or to the therapeutic agent [185].

Miltefosine has several adverse effects, most of which are self-limiting gastrointestinal reactions, in particular vomiting [141–143, 186], and it appears to have a low impact on liver and kidney function in dogs [139, 141]. Drug interactions, mainly associated with the inhibition of cytochrome c oxidase activity, have also been reported with the use of miltefosine in dogs [143].

The main side effects of allopurinol in the kidney are xanthinuria, xanthine tubular deposition, urolithiasis, and secondary urinary clinical signs [76, 140, 187]. In humans, elevated liver enzymes and gastrointestinal signs (diarrhea and nausea) have also been reported. In addition, there have been a few reports of cutaneous hypersensitivity, noncutaneous vasculitis, or drug interaction [188].

#### 20. When should anti-*Leishmania* treatment be stopped?

When the combined treatment in dogs of meglumine antimoniate or miltefosine for 1 month and allopurinol leads to clinical and clinicopathological improvement, the treatment should be continued with allopurinol alone for 12 months [158]. Its efficacy after administration for more than 1 year has not yet been demonstrated. After 12 months, this treatment could be withdrawn if the complete resolution of clinical and laboratory alterations has been achieved and when there is a marked reduction in both the number of antibodies and the parasite load [14, 29, 35].

Some dogs that are highly susceptible to leishmaniosis will never reach these improvements, while other dogs appear to have better control of the clinical signs and possibly the infection [21, 35, 189, 190]. Whether meglumine antimoniate or miltefosine can be safely administered to dogs for longer periods than described above needs further research.

#### 21. What is the best therapeutic approach if anti-*Leishmania* treatment is contraindicated?

Severe adverse effects caused by the administered drug or clinical resistance to the implemented treatment contraindicate the use of a particular anti-*Leishmania* treatment. If severe side effects associated with the recommended leishmanicidal drugs (meglumine antimoniate or miltefosine) are detected, the dog should be switched to the other treatment. A preexisting renal disease is not a contraindication for meglumine antimonate administration [6]. Note that with the use of miltefosine, it may take longer to obtain a clinical cure, the initial efficacy may be lower, and the percentage of relapses may be higher compared with dogs treated with meglumine antimoniate [78, 141, 143, 166, 174] (see also question 28).

For serious allopurinol-associated adverse effects in the kidney, such as massive xanthinuria, xanthine tubular deposition, or urolithiasis [140, 187], the first step is to decide whether the dog should still be treated with allopurinol, otherwise the treatment should obviously be stopped. If treatment is continued, urine specific gravity could be reduced by increasing water consumption, and a low-purine diet could be implemented to reduce xanthinuria [140, 143], with a review of the impact after approximately 4 weeks. An alternative to reduce xanthinuria is to use allopurinol (10 mg/kg, orally, just once a day) for 12 months; however, the impact of this dose reduction on its efficacy has not been assessed sufficiently [78]. Another option is to replace allopurinol with nucleotide analogs [191], or to use domperidone instead; however, these options appear to be without published evidence.

#### 22. Is prednisone or prednisolone always necessary in the treatment of dogs with leishmaniosis?

The use of glucocorticoids (prednisone or prednisolone) in managing CanL is still an open issue; however, robust evidence either to support or refute it is still lacking. Some authors advocate for their ability to ameliorate clinical signs and improve outcomes [192, 193]. However, other authors discourage their use owing to potential negative effects [194, 195].

Several studies have reported the beneficial effects associated with the use of glucocorticoids (above all, prednisone and prednisolone at variable doses) in dogs affected by leishmaniosis with clinical presentations likely caused by immune complex deposition [50, 193, 196, 197]. However, among the abovementioned studies, only two [192, 196] have investigated dogs with renal disease, but neither included a control group, which thus makes the results difficult to interpret. The remaining studies that have reported the beneficial effects of glucocorticoids in CanL involved a small number of dogs with arthritis [197], pustular dermatitis [198, 199], or hemostatic dysfunction [193].

One study did not support the use of glucocorticoids in dogs with different clinical problems due to leishmaniosis [50]. However, evidence against glucocorticoid use remains similarly limited. Adamama-Moraitou et al. [200] discouraged the use of prednisolone at immunosuppressive doses in dogs with leishmaniosis owing to the potential risk of promoting parasite replication. However, this did not actually occur in any of the dogs included in their study. In contrast, disease reactivation following prolonged treatment with glucocorticoids has been described in human patients and murine models [201, 202].

The IRIS group recommends the use of immunosuppressive drugs in dogs with active immune-complex glomerulonephritis, which could be the case in many dogs with leishmaniosis and renal disease [203]. This recommendation is based on the prediction that suppressing humoral immunity and the associated glomerular inflammatory response positively influence the progression, severity, and clinical outcome of the disease [204, 205].

The use of prednisone or prednisolone at an anti-inflammatory dosage (0.7–1 mg/kg orally once a day over a 3–10-day period) to reduce inflammation secondary to immune complex deposition rather than to decrease their formation and circulation is based only on expert opinions [6]. Once the decision to treat a dog with leishmaniosis with glucocorticoids has been made, the clinician should therefore discuss the potential benefits and risks with the dog’s owners. In all cases, considering their potential side effects, glucocorticoids should be administered cautiously, adjusting the doses and monitoring dogs closely throughout the treatment period.

#### 23. What is the role of immunotherapy in CanL?

Since the host immune system, especially a strong Th1 immune response, plays a crucial role in the outcome of CanL, enhancing the host defense mechanisms with immunotherapy might be beneficial. Immunotherapeutic products such as domperidone and dietary nucleotides with AHCC have been investigated in dogs with leishmaniosis [15, 158].

Domperidone is a dopamine D2 receptor antagonist, which causes a reversible increase in prolactin, and a subsequent rapid increase in CD4^+^ T lymphocytes  and cytokines such as IL-2, IFN-γ, and TNF-α [44]. Domperidone can be used both for preventing and treating CanL [44]. In dogs with leishmaniosis, although domperidone was able to induce a reduction in both clinical signs and number of serum antibodies [206], it is best used along with a recommended anti-*Leishmania* treatment protocol; the potential interaction between drugs should also be investigated [207].

Oral administration of dietary nucleotides with AHCC can reduce the rate at which leishmaniosis progresses from a clinically healthy infected status to clinical disease [208]. Dietary nucleotides are naturally present in food, especially in meat, fresh seafood, seeds, and dried legumes, and play a key role in modulating the immune response [44, 158, 209]. In fact, dogs that were fed an immune-modulating diet showed an increase in Treg population and a decrease in Th1 inflammatory response, in addition to a slight decrease in clinical signs of CanL [210]. AHCC is a standardized extract of cultured shiitake or *Lentinula edodes* mycelia that has antioxidant activity and increases the Th1 immune response associated with an increase in natural killer (NK) cells, T cells, B cells, and cytokines, such as IL-12 and TNF-α [44, 209].

#### 24. Is there any benefit in using immunochemotherapy in CanL?

Currently recommended anti-*Leishmania* treatment protocols are only effective in controlling the disease and might produce adverse effects. Overusing these protocols may also lead to drug resistance. Consequently, immunotherapy has generated a lot of interest [2, 6–8, 145]. Advances in immune response knowledge have led to a better understanding of CanL pathogenesis, enabling new treatments to be developed on the basis of immune system activation, often referred as immunotherapy. Immunotherapeutic agents directly or indirectly enhance the host’s natural defenses, thereby restoring the impaired effector functions or decreasing an excessive response by the host.

Combining immunotherapy with chemotherapy drugs (i.e., immunochemotherapy) results in a synergic action with activation of the immune system and direct action of the drugs against *Leishmania* [33, 44, 148, 149, 211–215]. Immunochemotherapy has shown different levels of effectiveness in a few published field studies. The administration of domperidone [179, 206, 216, 217] or dietary complements, such as nucleotides with AHCC [45, 158, 191, 208], combined with the recommended anti-*Leishmania* treatment, has shown promising results (Fig. 1).

However, immunochemotherapy protocols for CanL need to be standardized. Although an individual immunotherapy agent does produce a durable immune response [158, 179], combining immunotherapies could target multiple steps of leishmaniosis-associated immunity and perhaps achieve long-term effects.

Finally, before recommending the combination of several immunotherapies at the same time in dogs with leishmaniosis, more scientific evidence is needed to demonstrate its real clinical efficacy and safety.

#### 25. Could serology help clinicians plan the next leishmaniosis control?

Some studies report that a substantial and gradual decrease in the number of circulating *Leishmania*-specific antibodies correlates with clinical improvement. However, other studies argue that there is no correlation between the number of circulating antibodies and clinical status, as antibody quantification is not always useful for treatment monitoring [35, 76, 118, 218]. Antibodies may remain detectable chronically, but it is reasonable to repeat serology to assess their dynamics after at least 3–4 months from the start of treatment. Subsequently, for the same reason, serology can be repeated at 6 and 12 months after diagnosis. Later, if the number of antibodies remains high, considering the geographical area and transmission season, the next control should be closer, in 2–4 months, to inform clinical decisions. However, if the serology values remain moderately high, then the next control could be scheduled 4–6 months later. Finally, if the number of circulating antibodies is low or even zero, the next control can be in 6–12 months.

Dogs should not be retreated just because the serology is positive, even if the number of circulating antibodies is high or has increased. However, if dogs show: (i) clinicopathological abnormalities and/or clinical signs potentially associated with *active-infection* sickness, and (ii) the number of circulating *Leishmania*-specific antibodies is ≥ 3 dilutions (IFAT) or ≥ threefold (ELISA) (which is the cutoff value considered positive by laboratories), they might be considered clinically sick. Hence, at the time of a follow-up examination, serology can be considered as having worsened or improved if there are at least two dilutions above or below (IFAT), or a value that is twofold higher or lower (ELISA) than the previous result [3, 4, 7, 8, 11, 23, 24, 86].

#### 26. Why are dogs nonresponding or early relapsing despite the recommended anti-*Leishmania* treatment protocols?

Nonresponding refers to dogs with persistent clinical and/or clinicopathological abnormalities after being treated with recommended anti-*Leishmania* treatment protocols. However, early relapsing refers to dogs that, after experiencing clinical and clinicopathological remission, present with abnormalities attributable to leishmaniosis within a year after withdrawal of previous anti-*Leishmania* treatment.

Knowledge regarding relapses in CanL is still limited. However, there has been a decrease in the number of relapses and an increase in the duration of clinical stability following improvements in treatment protocols [4, 76, 111]. For example, from more than 70% of relapses within the first 6 months after treatment with only meglumine antimoniate [153] to approximately 20% of relapses within the first 2 years after treatment with the combination of meglumine antimoniate and allopurinol [158]. Therefore, an early relapse occurs when the dog suffers from leishmaniosis a second time or has an active infection during the first year after completing a recommended treatment protocol with an initial period of clinical improvement.

Unlike previous findings [2], the occurrence of comorbidities, inadequate immune response, and unresponsiveness to anti-*Leishmania* treatment represent major therapeutic challenges in CanL, and these conditions could potentially be associated with nonresponse or early relapse [43, 111].

Several comorbidities negatively impact the clinical management and outcome of CanL and could therefore be a key cause of nonresponding and early relapsing dogs. This is especially true in the case of infectious diseases, which can lead to overlapping clinical signs and interfere with the dog’s immune response, thus worsening the clinical picture and complicating the diagnosis [43].

The response to the recommended anti-*Leishmania* protocol treatments is strongly influenced by the immune response profile of the individual dog (Th1 versus Th2 type) as well as the quantity of CIC formed. Sudden clinical signs such as apathy, anorexia, pain, or vomiting may be observed shortly after initiating leishmanicidal therapy, sometimes within 3–5 days. This is a side effect associated with an increase in the quantity of CIC, which are key mediators of the tissue damage caused by *L. infantum*. In fact, following leishmanicidal treatment, an increase in circulating *Leishmania*-derived antigens can occur as the parasites are killed [219]. Depending on how the precipitin reacts [204], if the concentration of these antigens equals or slightly exceeds the number of circulating *Leishmania*-specific antibodies, CIC may form in higher quantities. Their subsequent attachment to vascular walls and the activation of the dog’s inflammatory response can lead to clinical worsening or a lack of improvement in treated dogs (see also question 22).

Although resistance to anti-*Leishmania* drugs in dogs remains rare, it may contribute to treatment failure in some cases [144]. For instance, genetic markers of resistance to allopurinol in *L. infantum* isolates from dogs with clinical disease relapse have been described [144, 178, 220–222]. Some genetic variants have been associated with resistance to antimonials [144, 223, 224]. Several genetic adaptations of *Leishmania*, especially in promastigotes, may lead to miltefosine resistance [144, 165, 224]. Finally, greater *L. infantum* resistance to miltefosine and amphotericin B after treating a dog with miltefosine plus allopurinol has been reported [165].

#### 27. What is the best strategy for nonresponding or early relapsing dogs?

Current evidence is still insufficient to draw definitive conclusions regarding the role of the abovementioned factors in clinical outcomes of CanL [4] (see also question 26). However, from a clinical point of view, the CLWG suggests several recommendations for nonresponding or early relapsing dogs. Although the clinical management is similar, it is important to differentiate between nonresponding and early relapsing dogs. Moreover, by itself, a positive serology does not identify a dog as nonresponding or relapsing (see also questions 5, 11, 12, 13, 25, and 28). In these dogs, the clinician should perform a complete clinical and laboratory re-evaluation [4, 11] (see also question 5). At the same time, it is necessary to rule out coinfections mimicking or overlapping leishmaniosis abnormalities (e.g., ehrlichiosis) or the presence of concomitant non-infectious diseases, and to establish the causative role of *Leishmania* by demonstrating the presence of the parasite (microscopic identification or qPCR) [225–228]. Regarding qPCR, samples should be obtained from lesions suspected to be caused by leishmaniosis or, if there are no such lesions, from blood (see also questions 5, 9, 10, and 28).

In nonresponding dogs, it is imperative to recheck that drugs, dose interval, and treatment length were correct and to verify the owner’s compliance. If active infection or sickness is confirmed, both in nonresponding and early relapsing dogs, treatment with an alternative protocol should be considered and possibly adding immunotherapy to improve the dogs’ immune response. However, in dogs with late relapses, the initial protocol can be repeated. Furthermore, *Leishmania* resistance to those drugs that are recommended to treat CanL, namely, meglumine antimoniate, miltefosine, and allopurinol, can be tested to optimize therapeutic decisions [144, 222].

### Follow-up and prevention

#### 28. What are the recommendations for monitoring seropositive non-infected, infected, and sick dogs?

Every clinical control should assess whether the dog’s leishmaniosis clinical stage has changed [6] since diagnosis or the previous control (Table 1). Depending on the specific clinical situation of each dog, a physical examination, CBC, biochemistry, SPE, urinalysis, and quantitative serology with or without parasitological evaluation by microscopy or qPCR may be necessary at each follow-up visit [11]. All these data help the clinician to decide (i) whether the therapeutic management needs to be modified for that particular dog and (ii) when another follow-up should be scheduled [1, 4, 9, 11].

In seropositive non-infected dogs (stage A), performing an annual physical examination, CBC, serum biochemical examinations (which could include several inflammatory markers and SPE), urinalysis, and serological testing to confirm whether to maintain the same clinical classification is recommended. Optimal interpretation of serology results requires testing to be undertaken close to the beginning of the sand fly season [2, 4, 11, 23, 24].

In infected dogs (stage B), untreated for leishmaniosis because they are not showing clinical signs or clinicopathological alterations, a physical examination should be performed along with CBC, serum biochemical examinations (which could include several inflammatory markers and SPE), urinalysis, and serology. However, the higher the number of *Leishmania*-specific antibodies in the previous control, the shorter the timeframe over which the next control should be assessed (see also question 25).

In sick dogs (stages C and D), it is best to perform a physical examination, CBC, and serum biochemical examination, which could include several inflammatory markers, SPE, and urinalysis [6, 16, 86, 88]. This examination should take place at the end of the leishmanicidal treatment, at the end of meglumine antimoniate administration [118, 157], or 3–4 weeks after miltefosine has been terminated [145, 229]. These assessments, including quantitative serology for leishmaniosis, can then be repeated every 3–4 months during the first year after leishmanicidal treatment.

Note that high quantity of antibodies (see also questions 25 and 26) or detection of *Leishmania* with qPCR in bone marrow are not sufficient to diagnose that a dog is suffering from leishmaniosis or has an active infection because, generally, dogs remain chronically infected with a low quantity of parasites. The microscopic visualization of *Leishmania* in any type of sample or positive qPCR in lesions or blood indicates that the dog has relapsed and therefore needs anti-*Leishmania* retreatment [2–4, 6, 11, 14].

The CLWG recommendations, outlined above on monitoring frequency and tests to be performed during each follow-up visit, reasonably vary depending on the health status of the dog, the owners’ compliance, and the clinical decisions of the veterinarian.

#### 29. What are the recommendations for the prevention of CanL in the environment?

The most efficient way to prevent CanL is a combined approach based on optimizing environmental conditions, reducing infection and transmission, and controlling disease development [1, 158, 230].

Controlling phlebotomine vectors in the environment is difficult owing to the biology and ecology of these insects. In fact, insecticides and indoor residual spraying (IRS) have been used in, for example, outbreaks of zoonotic visceral leishmaniosis in humans [1, 231]. In this case, IRS consists of spraying long-acting insecticides on the walls and roofs of houses and pet shelters and is an efficient method of controlling leishmaniosis. IRS has been used in human dwellings, above all to control mosquitoes or other insects, and consequent control of sand flies is only coincidental [1, 231].

The most used insecticides for leishmaniosis vector control are organophosphate (chlorpyrifos-methyl), carbamate (propoxur), and pyrethroid (α-cypermethrin, cypermethrin, deltamethrin, and l-cyhalothrin). The effectiveness of IRS may depend on the environment as well as on the total treated area [1]. Thus, the control of sand flies and, in turn, leishmaniosis is more effective in urban settings, where every house and pet shelter is likely to be treated, than in rural areas.

The gold standard synthetic insect repellent is *N*-diethyl-3-methylbenzamide (DEET), which is highly effective against hematophagous insects, including *Leishmania* vectors, and has been in use for over half a century [232]. Owing to the detrimental effects of synthetic insecticides and repellents on the environment, their acceptance among society is decreasing. Consequently, researchers have investigated a variety of natural oils extracted from plants. For example, allspice, amyris, catnip, neem, Mexican oregano, peppermint, and tea tree appear to generate repellent behavior in sand flies [233–235]. However, they are not sufficiently effective for use in the environment or for direct use on animals.

#### 30. What are the recommendations for the prevention of infection through sand fly bites?

The second step for the prevention of CanL entails controlling infection and transmission. The focus is on preventing the sand fly vector from biting receptive hosts by minimizing exposure and controlling the vector population. In veterinary medicine, the use of repellents, such as pyrethroids (e.g., deltamethrin, flumethrin, or permethrin) in different formulations (e.g., impregnated collars or spot-on formulations) is the first-line approach to reduce the risk of phlebotomine sand flies biting dogs and therefore the risk of *Leishmania* spp. transmission to animal species, including humans [230, 236].

Repellents induce killing and antifeeding effects, and late mortality of sand flies occurs after encountering the parasiticide on the fur or the skin of the treated host [237]. Depending on their formulation and the correct use, pyrethroids have shown different levels of efficacy under laboratory and field conditions, which typically last from weeks to 1 year. The use of pyrethroid formulations against *L. infantum* infection in dogs varies according to the geographical areas and specific epidemiological scenario. While dogs living in nonendemic regions should be treated when traveling to endemic areas, those living in endemic areas should be treated during the sand fly season in that specific geographical area [238]. However, with climate change, all-year-round prevention may be preferable. By virtue of their systemic insecticidal activity and proven efficiency in reducing the number of different species of phlebotomine sand flies in specific foci, isoxazolines could be used to complement pyrethroids as a control strategy for human and canine leishmaniosis prevention in endemic areas [236, 239].

#### 31. What are the recommendations for the prevention of non-arthropod infection?

CanL is primarily transmitted through the bites of infected female phlebotomine sand flies (vectorial transmission). However, there are also other less common nonvector modes of transmission, such as in utero infection, exposure to parasites within blood products, and venereal transmission [226, 240–243]. Additionally, direct dog-to-dog transmission through bites or wounds has been suspected as a possible reason for the spread of *Leishmania* in nonendemic areas [244, 245]. The period of incubation is similar for each type of transmission, ranging from 3 months to 7 years [241, 244–247].

Since venereal and vertical transmission may have epidemiological significance in the dissemination and maintenance of the disease, especially in the absence of the biological insect vector, regular testing of breeding dogs before mating is essential [240, 242, 243, 248–252].

Blood transfusion is another potential source of infection, and thus, dogs should be carefully screened for *Leishmania* before donating blood. This aspect is crucial because an infected dog may be asymptomatic but still capable of transmitting the parasite to a recipient dog through transfusion [253–256].

Although these nonvector transmission modes probably only play a marginal role in the epidemiology of CanL, raising awareness among dog owners and veterinarians is key to preventing the spread of CanL [1, 7, 8]. By ensuring regular testing, promoting responsible breeding, and taking precautions with blood transfusions, the risk of nonvector transmission can be significantly minimized and help to control the spread of this disease in dogs.

#### 32. What are the risk factors contributing to the spread of CanL?

CanL by *L. infantum* is prevalent globally, except in Oceania [18, 190, 238]. The distribution of infection overlaps that of the sand fly vectors. Since its introduction to the New World via infected dogs from Europe [257], *L. infantum* has found many suitable vectors (e.g., phlebotomine sand flies of the genus *Phlebotomus* and *Lutzomyia* in the Old and New Worlds, respectively) and vertebrate hosts.

In the last few decades, this protozoon has extended its distribution to areas where it was not previously endemic, such as northern Italy and Spain, and Central and Northern Europe, as well as northern Argentina and the USA [258]. Among the many drivers for the increasing distribution of CanL is the expansion of sand fly vectors and their colonization of new ecological niches, as well as the global increase in mean temperatures.

Human and animal movements, deforestation, and urbanization, among other factors, have contributed to the establishment of new stable foci of infection niches [238, 259, 260]. As cases of canine infection can occur where sand flies are active, in most endemic areas, this can occur all year round.

Although dogs represent the main reservoirs of *L. infantum* worldwide, other mammals (e.g., rodents, marsupials, nonhuman primates, and carnivores) may act as reservoirs, depending on many ecological factors, such as sand fly vector density, species composition, and blood-feeding preferences, together with the length of the transmission season [238, 261]. For example, though cats, dogs, and humans are a source of *L. infantum* infection for phlebotomine sand flies in Europe, both the black rat (*Rattus rattus*) and the Iberian hare (*Lepus granatensis*) may be infecting sand flies, possibly maintaining the infection when the principal reservoirs are lacking [262, 263].

#### 33. What are the current recommendations for the immunotherapy of dogs against leishmaniosis?

Because prevention of infection is not 100% effective, the third step is to seek to prevent the development of the disease or decrease the severity of clinical signs in an infected dog [44, 211, 212]. This type of prevention alone is much less effective than preventing infection, but combined with the use of pyrethroids, it could help to increase prevention effectiveness [1, 8, 44, 135, 264, 265]. This is achieved by activating the dog’s immune system to keep the parasite load very low and thus prevent the development of clinical signs or analytical alterations [15, 158, 208, 266].

Activation or modulation of the immune system through immunotherapy is based on the use of vaccines, drugs such as domperidone, or nutraceuticals such as dietary nucleotides with AHCC. The immunotherapy protocol recommended for leishmaniosis is different for each dog and will depend on multiple factors, such as whether or not the dog lives in an endemic area, the level of awareness of the owners, or label recommendations for each product [44, 211, 212]. However, it is up to the attending clinician to determine whether vaccines should be administered for the prevention of leishmaniosis in an individual dog [267]. A vaccine may thus be potentially used in infected dogs as immunotherapy [267] or even in sick dogs as immunochemotherapy [215]; however, more scientific evidence is needed to confirm this use. Note that the vaccines currently on the market are only registered to be used in healthy seronegative dogs [136, 268].

#### 34. What do we currently know about vaccines for CanL?

Although more than 20 years have passed since the first vaccine against CanL was launched, concerns about its practical utility remain [269] (see also questions 14 and 33). Current evidence indicates that none of the available vaccines (Letifend and Neoleish in Europe and Leish-Tec in Brazil) are totally effective in preventing the infection in vaccinated dogs, as they only reduce, rather than eliminate, the risk of developing clinical disease [261, 268–270]. Owners should thus be aware that after vaccination, the development of clinical disease is still possible. As the potential role of vaccinated dogs as reservoirs of the disease cannot be excluded, the use of repellents in these dogs is always recommended [261, 268].

In the best scenario, at least 1 month is required for immunity induction, and thus vaccination does not seem appropriate for dogs traveling to endemic areas for short stays. Although beneficial effects with vaccine administration in infected and sick dogs (when used as an adjuvant to the leishmanicidal therapy) have been reported [270, 271], evidence is still sparse. As this approach represents an off-label use, it cannot be recommended.

Finally, some clinicians combine the vaccine with other immunotherapeutic agents, such as domperidone or nucleotides with AHCC [44]; however, it is still not understood whether or not these measures add an extra preventive effect.

## Conclusions

Canine leishmaniosis continues to attract significant interest worldwide owing to its geographical diffusion and the increasing number of dogs being identified earlier as infected or sick owing to the greater understanding and availability of diagnostic tests. Veterinary practitioners should therefore be aware of the challenges involved in managing this disease. The recommendations presented in this document update and summarize the current state of knowledge regarding the clinical presentation, diagnosis, treatment, monitoring, prognosis, and prevention of this disease.

## Data Availability

Data supporting the main conclusions of this study are included in the manuscript.

## Abbreviations

AHCC: Active hexose dietary compound
APP: Acute-phase proteins
aPTT: Activated partial thromboplastin time
AT: Antithrombin III
CanL: Canine leishmaniosis
CBC: Complete blood count
CIC: Circulating immune complexes
CKD: Chronic kidney disease
CLWG: Canine Leishmaniosis Working Group
CRP: C-reactive protein
DEET: *N*-diethyl-3-methylbenzamide
DNA: Deoxyribonucleic acid
ELISA: Enzyme-linked immunosorbent assay
FDP: Fibrin/fibrinogen degradation products
GGT: Gamma-glutamyl transferase
Hp: Haptoglobin
ICT: Immunochromatographic tests
IFAT: Immunofluorescence antibody test
IFN: Interferon
IgG: Immunoglobulin G
IL: Interleukin
IRS: Indoor residual spraying
IRIS: International Renal Interest Society
*L. braziliensis*: *Leishmania braziliensis*
*L. infantum*: *Leishmania infantum*
*L. tarentolae*: *Leishmania tarentolae*
NAG: *N*-acetyl-beta-d-glucosaminidase
NGAL: Neutrophil gelatinase-associated lipocalin
NK: Natural killer
PON-1: Paraoxonase
PT: Prothrombin time
qPCR: Quantitative polymerase chain reaction
SAA: Serum amyloid A
SDS–AGE: Sodium dodecyl sulfate–agarose gel electrophoresis
SG: Specific gravity
SPE: Serum protein electrophoresis
SLA: Soluble *Leishmania* antigen
Th: Lymphocytes T helper
TNF: Tumor necrosis factor
uGGT: Urinary gamma-glutamil transferase
UPC: Urine protein-to-creatinine ratio
